# Appendicitis with perforation caused by metal dental graft: A case report

**DOI:** 10.1097/MD.0000000000042056

**Published:** 2025-04-04

**Authors:** Yinmin Sun, Shiqi Liu, Yufeng Li, Dongwen Quan, Siqi Li

**Affiliations:** a The Second Clinical Medical School, Shaanxi University of Chinese Medicine, Xianyang, Shaanxi Province, China; b Department of Neonatal Surgery, Xi’an Children’s Hospital Affiliated Xi’an Jiaotong University, Xi’an, Shaanxi Province, China; c Department of Pediatric Surgery, Guilin Maternal and Child Health Hospital, Guilin City, China.

**Keywords:** abdominal X-ray, acute appendicitis, appendiceal perforation, CT scan, foreign body, teeth

## Abstract

**Rationale:**

Under normal circumstances, foreign objects in the digestive tract can be expelled naturally through the anus via the digestive tract’s peristalsis mechanism. In clinical practice, foreign bodies ingested orally seldom pass through the cecum into the appendiceal lumen, potentially leading to secondary appendicitis or even perforation – a highly infrequent but severe complication. This article aims to share our experience and insights in managing such rare cases, exploring diagnostic pathways and treatment options, and serving as a reference for colleagues.

**Patient concerns:**

A 57-year-old male patient may have accidentally ingested a tooth graft, resulting in right lower quadrat pain. Based on the physical examination and imaging findings, the likelihood of AA with perforation is high.

**Diagnoses:**

Acute appendicitis (AA) with perforation.

**Interventions:**

Laparoscopic appendectomy (LA) and removal of foreign bodies.

**Outcomes:**

After surgical treatment, the patient recovered well, and there were no recent complications such as wound infection, incisional hernia, and massive hemorrhage.

**Lessons:**

In the diagnosis of appendicitis caused by foreign bodies, clinical history, physical examination, and imaging are crucial to the diagnosis of the disease. Once diagnosed, surgery should be taken quickly. LA offers several advantages, including minimal trauma, excellent visualization, precise treatment outcomes, and swift patient recovery. Therefore, we recommend it as the preferred treatment for appendiceal perforation caused by foreign bodies.

## 1. Introduction

AA is a common cause of surgical acute abdomen, with an annual incidence of approximately 0.1% worldwide. It usually presents with periumbilical pain and progresses to the right lower quadrat (RLQ). The diagnosis of AA is based on a comprehensive analysis of history, physical examination, laboratory evaluation, and imaging.^[1]^ The cause of AA is usually thought to be caused by the obstruction of the appendix cavity, the common cause is the proliferation of lymphatic follicles, in addition to the accumulation of feces, foreign bodies, calculi, bacterial invasion, parasites, benign and malignant tumors. Lymphoid follicular hyperplasia is considered to be the main cause of AA in young adults, while luminal obstruction by calculi or mass is more common in elderly patients.^[2]^ Appendicitis caused by foreign bodies is a relatively uncommon condition, often leading to complications such as perforation, rupture, and infection of the appendix. The literature documents several cases of acute appendicitis (AA) resulting from various foreign bodies obstructing the appendix lumen, including needles, toothpicks, seeds, fish bones, and others.^[3]^

Reviewing the existing literature, it can be found that the main causes of appendicitis caused by odontogenic foreign bodies are incorrect swallowing of self-broken teeth, dental crowns, dentures, dental caries with metal fillings, etc.^[4–8]^ If the patient accidentally ingests such odontogenic foreign bodies and deposits them in the appendiceal lumen, it may lead to obstruction of the appendiceal lumen and secondary infection. Here, we document in detail the case of a 57-year-old male patient. The patient accidentally swallowed a dental implant made of a metal abutment and a metal artificial tooth. The foreign body blocked the appendiceal lumen as it passed through the digestive tract, subsequently inducing AA and eventually leading to the perforation of the appendix.

## 2. Case presentation

A male patient, 57 years old, was admitted to the hospital due to “RLQ pain for 4 days with acute exacerbation for 1 day.” The patient stated that he might have swallowed a dental graft by mistake 4 days ago, and then had intermittent pain in the RLQ. Recently, the pain gradually intensified, accompanied by nausea and retching during the pain. Since the onset of the disease, the patient has had poor mental state and sleep, poor appetite, normal urination, and no obvious change in weight. Physical examination: The McBurney point in the RLQ has obvious tenderness, positive rebound pain, muscle tension, negative Murphy sign, and weakened bowel sounds. Body temperature, 38.1 °C; blood pressure, 135/76 mm Hg; heart rate, 95 beats per min; respiratory rate, 20 breaths per min. Previous health, no chronic disease, infectious disease and family genetic history. Laboratory examination: White blood cell count 17.17 × 10^9^/L, neutrophils 88%, C-reactive protein 30.15mg/L.

**Figure 1.**
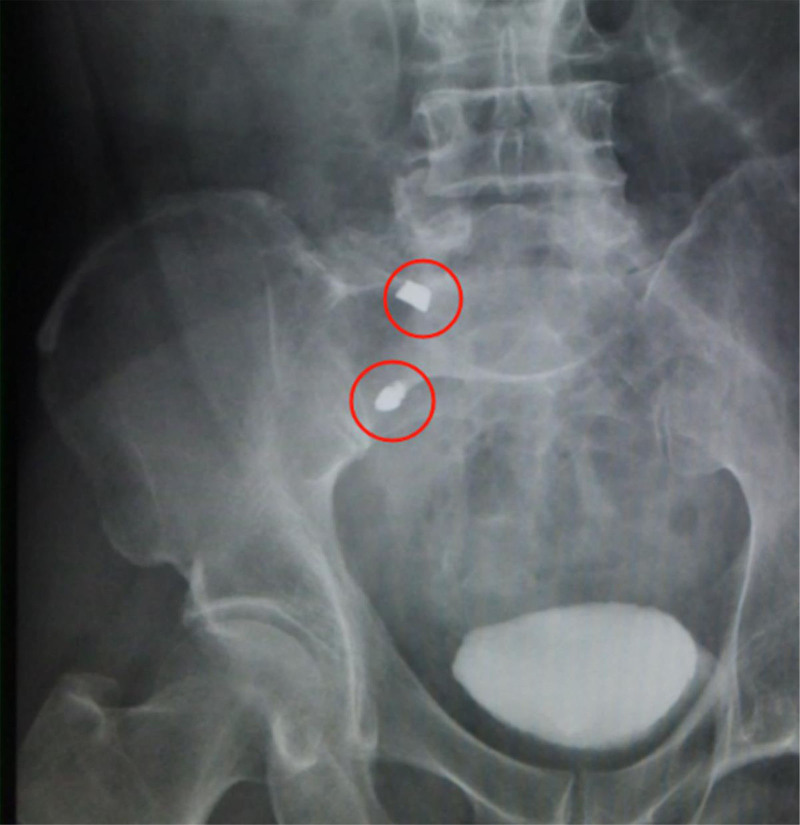
Abdominal X-ray showing 2 metallic objects.

**Figure 2.**
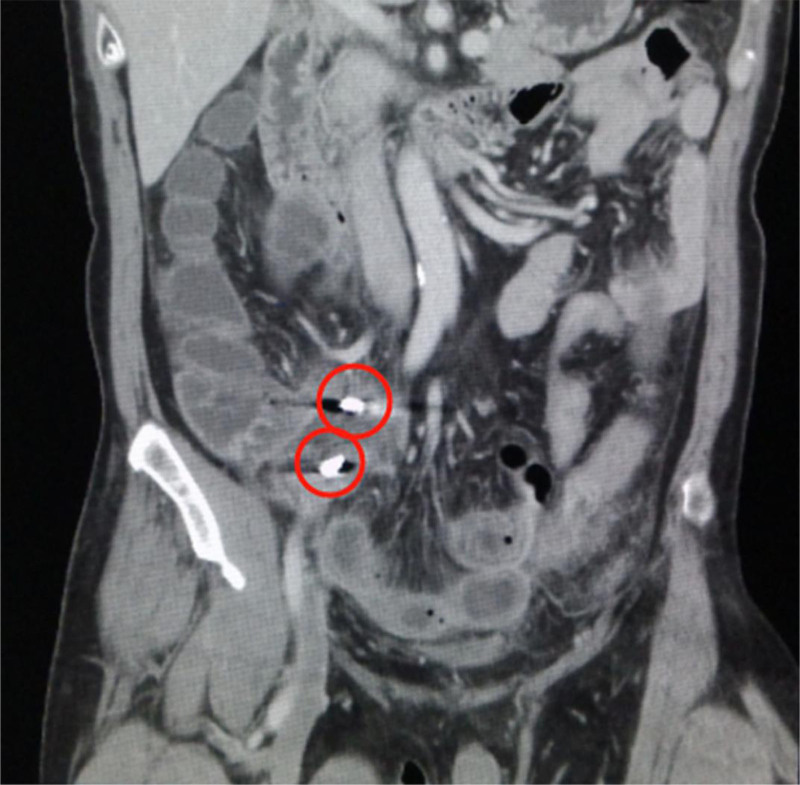
Computed tomography scan showing metallic objects around the appendix.

Upon admission, the patient underwent both a plain abdominal X-ray (Fig. 1) and a computed tomography (CT) scan (Fig. 2). Imaging revealed that the tissue around the ileocecal part was edema, there were ascites in the abdominal cavity, and there were 2 high-density metal shadows, the size of which were 1.0 × 0.8 cm and 1.0 × 0.6 cm, respectively. The suspected ingested tooth graft consists of a metal abutment and a metal artificial tooth, and it is not yet possible to determine whether the metal foreign body is located inside or outside the bowel. Following clinical assessment and abdominal X-ray and CT imaging, physical examination revealed signs of peritoneal irritation, and the patient’s abdominal pain progressively intensified. A diagnosis of AA complicated by perforation was confirmed. Given the critical nature of the patient’s condition, an appendectomy was carried out following a confirmed diagnosis. Prior to surgery, intravenous fluids were administered to restore hydration and correct electrolyte imbalances, thereby preventing dehydration. Prophylactic antimicrobial therapy with Cefoperazone/Sulbactam was also instituted.

Surgical Procedure: General anesthesia was used and the patient was placed in a supine position. The surgical area should be disinfected and covered with sterile sheets. Monitor vital signs (blood pressure, heart rate, oxygen saturation). Pneumoperitoneum was established with Veress needle through the umbilical cord, trocar was inserted, and the pressure was maintained at 12 to 15 mmHg. Three-hole laparoscopic surgery was selected, and the umbilical hole (Trocar 1) was 10mm for laparoscopic implantation. RLQ above McLeod point (Trocar 2): 5mm for operation; Right upper abdomen (Trocar 3): 5mm for auxiliary operation. Trocar 1 was implanted into the laparoscope to explore the abdominal cavity, confirm the location of the appendix, observe its shape and surrounding tissue status, and look for metal foreign bodies. You can see that one of the metal foreign bodies is surrounded by inflammatory tissue around the appendix, and the other is outside the appendix lumen. Trocar 2 and 3 were used to insert operating instruments to separate and remove the metal foreign body. The root of the appendix was closed with an absorbable clamp. After no bleeding was confirmed, the appendix was removed with an electric coagulation hook and the appendix specimen was taken out. Carefully examine the incision and other areas of the abdominal cavity to ensure that there is no bleeding or residue. Use an anti-adhesion solution to flush the abdominal cavity to prevent tissue adhesion. After carefully checking the incision for foreign bodies, the Trocar incision was closed layer by layer and the skin was closed with absorbable sutures. Pneumoperitoneum was removed and the patient’s vital signs were observed to be stable.

Postoperative management: The patient was transferred to the recovery room and subsequently to the ward once vital signs were stable, with continuous monitoring of vital parameters. Postoperatively, Cefoperazone/Sulbactam therapy was continued for 4 days, along with wound care and symptomatic management, until the patient’s body temperature and white blood cell count returned to normal ranges. No complications, including wound infection, incisional hernia, or significant hemorrhage, were observed.

## 3. Discussion and conclusions

AA is a common surgical disease that is usually associated with a variety of factors, including lymphadenosis, fecalith, bacterial infection, parasitic infection, tumor or, in rare cases, obstruction of the appendix cavity caused by ingestion of foreign bodies.^[2]^ Appendicitis caused by foreign bodies is a relatively rare condition because most mistakenly swallowed foreign bodies can pass through the normal channels of the digestive tract and eventually pass out of the body without causing any complications or requiring surgical intervention. Previously, it was reported that various foreign bodies such as needles, toothpicks, seeds, teeth, fish bones, etc caused AA.^[3,9]^ In theory, when the foreign body ingested by the human body is metal or other high-density substances, the movement of these foreign bodies may be limited in the cecum due to their large weight and inability to be digested and absorbed in the gastrointestinal tract. However, the lumen of the appendix is thin and the opening is narrow. Once these foreign bodies enter the lumen of the appendix, the peristalsis of the appendix is not enough to push them to the colon, resulting in the retention of foreign bodies in the lumen of the appendix, and then induce AA. In severe cases, it can cause complications such as appendiceal perforation or abdominal infection.^[10]^

Appendiceal perforation caused by foreign bodies is a rare clinical condition, and its pathogenesis is closely related to daily eating habits, swallowing or accidental ingestion of food foreign bodies. In the case of this study, the patient developed the disease due to accidental ingestion of a metal tooth graft, which consisted of a metal abutment and an artificial tooth. These 2 metal foreign bodies deposited in the lumen of the appendix, causing lumen obstruction, which subsequently triggered an inflammatory reaction and eventually developed appendiceal perforation. In the diagnosis of appendicitis caused by foreign bodies, clinical history, physical examination, and imaging examination are essential for the diagnosis of the disease. Once diagnosed, operative management (OM) should be taken quickly.^[4,5,10]^

Recent literature has reported that the comparison of OM and non-operative management (NOM) of appendicitis is controversial. It has been suggested that laparoscopic appendectomy (LA) is effective in the treatment of AA with a low cost and recurrence rate. However, there is still a 60% to 70% chance of avoiding OM, and appropriate antibiotic treatment can be selected for simple and uncomplicated appendicitis.^[11]^ Conservative treatment of uncomplicated appendicitis with antibiotics may reduce surgical complications and hospitalization. However, several risk factors have been reported to be associated with the recurrence of appendicitis in patients NOM, including the presence of a fecalith in the appendix and an appendix larger than 1cm in diameter on imaging.^[12]^ There are numerous reports on the conservative management of AA, and the use of antibiotics in the treatment of uncomplicated appendicitis is effective and reduces the short-term hospitalization rate.^[11,12]^ However, among patients initially treated with antibiotics, recurrence rates were reported to be relatively high, with 29% requiring surgery within 90 days, 27.3% within 1 year, and 39.1% within 5 years. Studies have shown that OM does not increase the recurrence rate or the risk of complications of appendicitis.^[13]^ Appendectomy is the standard treatment for AA and can be performed either open or laparoscopic. Studies have shown that LA is superior to open appendectomy because it reduces patient pain intensity, wound infection rates, length of hospital stay, and postoperative quality of life.^[14]^ Although there are a large number of reports on the NOM of AA in the current research, LA is still the gold standard for most treatments, especially for appendicitis caused by foreign bodies, and timely LA is the preferred treatment.^[9,15]^

Although most ingested foreign bodies can pass through the digestive tract smoothly, complications such as perforation, obstruction, and bleeding are more likely to occur for sharp, stiff, thin, pointed, long, and heavier objects. In the diagnosis of AA caused by foreign bodies, the clinical symptoms are similar to typical AA. Therefore, we should focus on asking the patient’s medical history and make a definite diagnosis combined with the results of imaging examination and laboratory examination.^[3]^ When there are foreign bodies in the appendix, especially high-density foreign bodies, plain abdominal X-ray and CT scan can show the inflammation and state of the appendix, which has significant significance for clinical diagnosis.^[5,16]^ As in the case of this patient, the history of accidental ingestion of foreign bodies and imaging revealed 2 highly transparent foreign bodies in the appendix region, which provided clear guidance for OM. As reported by Sudeshna Sar,^[17]^ the patient presented with right iliac fossa pain. CT scan showed a mass of the appendix with a small abscess and a linear opaque foreign body. Initially, conservative management was instituted, resulting in symptom resolution. However, a few months post-surgery, the patient re-presented with recurrent symptoms and elevated inflammatory markers in the right iliac fossa. A follow-up abdominal CT scan demonstrated a persistent abscess with foreign bodies at the same site. The patient subsequently underwent appendectomy, abscess drainage, and foreign body removal. Surgeons should be vigilant when foreign bodies are identified in the appendix, as these cases necessitate prompt surgical intervention and formal appendectomy.

Appendicitis caused by foreign bodies is rare, but it has unique clinical characteristics and diagnostic basis. This case of AA with perforation caused by accidental ingestion of metal dental graft highlights the importance of detailed medical history and reasonable use of imaging. Once the diagnosis is confirmed, LA is the first choice. During the operation, it is crucial to completely remove foreign bodies, control infection and ensure complete removal of the appendix.

## Acknowledgments

We would like to express our gratitude to the patient for granting permission to use their clinical data in this paper and for the publication of this research.

## Author contributions

**Conceptualization:** Yufeng Li.

**Data curation:** Yinmin Sun, Shiqi Liu, Dongwen Quan, Siqi Li.

**Formal analysis:** Yinmin Sun, Shiqi Liu, Yufeng Li, Dongwen Quan.

**Writing – original draft:** Yinmin Sun, Dongwen Quan.

**Writing – review & editing:** Shiqi Liu.
